# *fog-2* and the Evolution of Self-Fertile Hermaphroditism in *Caenorhabditis*

**DOI:** 10.1371/journal.pbio.0030006

**Published:** 2004-12-28

**Authors:** Sudhir Nayak, Johnathan Goree, Tim Schedl

**Affiliations:** **1**Department of Genetics, Washington University School of MedicineSt. Louis, MissouriUnited States of America; University of California at BerkeleyUnited States of America

## Abstract

Somatic and germline sex determination pathways have diverged significantly in animals, making comparisons between taxa difficult. To overcome this difficulty, we compared the genes in the germline sex determination pathways of Caenorhabditis elegans and *C. briggsae,* two *Caenorhabditis* species with similar reproductive systems and sequenced genomes. We demonstrate that C. briggsae has orthologs of all known C. elegans sex determination genes with one exception: *fog-2*. Hermaphroditic nematodes are essentially females that produce sperm early in life, which they use for self fertilization. In *C. elegans,* this brief period of spermatogenesis requires FOG-2 and the RNA-binding protein GLD-1, which together repress translation of the *tra-2* mRNA. FOG-2 is part of a large C. elegans FOG-2-related protein family defined by the presence of an F-box and Duf38/FOG-2 homogy domain. A *fog*-2-related gene family is also present in *C. briggsae,* however, the branch containing *fog-2* appears to have arisen relatively recently in *C. elegans,* post-speciation. The C-terminus of FOG-2 is rapidly evolving, is required for GLD-1 interaction, and is likely critical for the role of FOG-2 in sex determination. In addition, *C. briggsae gld-1* appears to play the opposite role in sex determination (promoting the female fate) while maintaining conserved roles in meiotic progression during oogenesis. Our data indicate that the regulation of the hermaphrodite germline sex determination pathway at the level of FOG-2/GLD-1/*tra-2* mRNA is fundamentally different between C. elegans and *C. briggsae,* providing functional evidence in support of the independent evolution of self-fertile hermaphroditism. We speculate on the convergent evolution of hermaphroditism in *Caenorhabditis* based on the plasticity of the C. elegans germline sex determination cascade, in which multiple mutant paths yield self fertility.

## Abstract 2

A comparison of sex determination genes in *C. elegans* and *C. briggsae* provides evidence in support of the convergent evolution of self-fertile hermaphroditism in the *Caenorhabditis* clade

## Introduction

Sex determination is an ancient and universal feature in metazoans. In spite of this, comparison of distantly related species such as Caenorhabditis elegans and Drosophila melanogaster has revealed little about the evolution of the complex pathways that mediate the sexual fate decision in the soma and germline [1,2,3]. This is likely due to the combination of gross morphological, functional, and behavioral dissimilarity and extensive sequence divergence. Thus, if we wish to clarify the etiology of diverged sex determination pathways, an alternative approach is required.

One approach is to perform comparative analysis of sex determination genes in species separated by sufficient evolutionary time to allow for changes in pathway components yet retain comparable somatic and germline morphology and function. The clade containing C. elegans and C. briggsae represents an ideal case for this type of study, as the sex determination pathway has been well studied in C. elegans and an abundance of sequence information is available for both species [4,5].

C. elegans and *C. briggsae,* while sharing very similar germline and somatic morphology, are separated by approximately 100 million years and are members of a clade that employs multiple mating systems [5,6,7,8,9,10]. C. elegans and C. briggsae are self-fertile hermaphrodites that maintain males at a low frequency (androdioecious), whereas the morphologically similar C. remanei and *C.* sp. CB5161 are obligate female/male (gonochoristic) species [6,7,10]. Phylogenetic analysis of the four closely related *Caenorhabditis* species suggests that self-fertile hermaphroditism has evolved independently in C. elegans and C. briggsae from an ancestral male/female state [10,11]. Importantly, a transition in mating system from female/male to hermaphroditic (or hermaphroditic to male/female) requires that one or more changes in the sex determination pathway have occurred.

C. elegans and *C. briggsae,* like many other animals, have two sexes specified by the ratio of X chromosomes to sets of autosomes [8,12,13]. In both species, XX animals are somatically female while the germline is hermaphroditic. Self fertility is achieved by a transient period of spermatogenesis beginning in the third larval (L3) stage before the organism switches to the production of oocytes in the L4 stage, which continues throughout adulthood [14,15]. In both species, XO males begin sperm production in the L3 stage and continue spermatogenesis throughout their reproductive lives [14,16,17].

**Figure 1:**
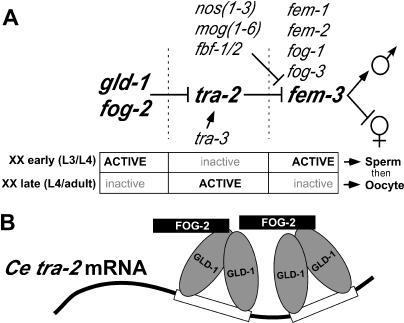
The C. elegans XX Hermaphrodite Germline Sex Determination Pathway (A) Genetic pathway for gene activity, where arrows represent positive regulation and bars represent negative regulation. The key genes *tra-2* and *fem-3* and the upstream regulators of *tra-2* that are the focus of this work, *fog-2* and *gld-1,* are in large bold font. The upstream genes *fog-2* and *gld-1,* which are key regulators of *tra-2* and addressed in this work, are also in large bold font. The gene activities at each level in the hierarchy are indicated below as “ACTIVE” in bold or “inactive” in grey. In L3 and L4 hermaphrodites the activities of *fog-2* and *gld-1* are high, leading to repression of *tra-2* activity (also see [B]) and the de-repression of *fem-3,* resulting in the onset of spermatogenesis. In L4 and adult hermaphrodites the activity of *fog-2* and *gld-1* are low, leading to high *tra-2* activity and the repression of *fem-3,* resulting in oogenesis. The shift in *tra-2*/*fem-3* balance allows for the switch from spermatogenesis to oogenesis in an otherwise female somatic gonad in the hermaphrodite. (B) C. elegans FOG-2/GLD-1/*tra-2* mRNA ternary complex. Current data indicates that FOG-2 and GLD-1 are required for the translational repression of the *tra-2* mRNA [25]. GLD-1 binds as a dimer to the *tra-2* mRNA 3′UTR at two 28 nucleotide direct repeat elements (TGE/DRE, blocks) and FOG-2 makes contact with GLD-1 [32,34]. All three components are required for the proper specification of hermaphrodite spermatogenesis.

A major determinant of germline sexual fate in C. elegans is the relative activity of two key regulators: *tra-2,* which promotes the female fate (oocyte), and *fem-3,* which promotes the male fate (sperm) [18,19] (Figure 1A). The activities of *tra-2* and *fem-3* must be regulated in both males and hermaphrodites to allow spermatogenesis to occur, however the mechanisms by which this regulation occurs differs between the two sexes. In males, *her-1* represses *tra-2* feminizing activity and raises the relative level of *fem-3* activity so that spermatogenesis is continuous [20,21]. Since null mutations in *her-1* have no effect on hermaphrodites and *her-1* is not expressed in XX animals, a different mechanism is used to allow for the transient production of sperm [22,23].

Self fertility in C. elegans hermaphrodites is achieved by an early period of spermatogenesis followed by a later period of oogenesis (Figure 1A). The promotion of spermatogenesis during the L3 stage (early) is achieved by translational repression of the *tra-2* mRNA mediated by *gld-1* (“defective in germline development”) and *fog-2* (“feminization of germline”)[24,25] (Figure 1A and 1B). The transient reduction in the level of *tra-2* feminizing activity raises the relative level of *fem-3* masculinizing activity to promote spermatogenesis (Figure 1A). Later in L4 and adult animals, oogenesis is promoted by relieving the *fog-2/gld-1*-mediated repression of *tra-2* feminizing activity combined with repression of *fem-3* masculinizing activity by *mog-1* to *mog-6, fbf-1 and fbf-2,* and *nos-1* to *nos-3* [18,19,26].

Central to this work are the genes *fog-2* and *gld-1. fog-2* is required for hermaphrodite, but not male, spermatogenesis in *C. elegans,* as XX animals that lack *fog-2* produce only oocytes, resulting in functional females, whereas XO males are unaffected [27]. Similarly, loss-of-function mutations in *gld-1* result in the feminization of the hermaphrodite germline without affecting males [28,29]. Both *fog-2* and *gld-1* are germline-specific regulators of sexual fate, since they do not appear to be expressed in the soma, and null mutations in either gene do not affect somatic sexual fate [25,27,28,29,30].

*C. elegans gld-1* is a germline-specific tumor suppressor that is indispensable for oogenesis [28,29] and encodes a conserved KH-type RNA-binding protein [30]. GLD-1 is a translational repressor that binds to multiple mRNA targets [31], including *tra-2,* where it binds as a dimer to each of two *tra-2* and GLI elements (TGEs) present on the 3′ untranslated region (UTR) of the *tra-2* mRNA [24,32] (Figure 1B). Deletion of the *tra-2* TGEs results in a loss of GLD-1-mediated translational control and feminization of the germline, such that only oocytes are produced [20,25,33,34].

C. elegans FOG-2 was identified as a GLD-1-interacting protein with a structure similar to canonical F-box proteins; it has an N-terminal F-box and a C-terminal protein–protein interaction domain. In the case of FOG-2 the putative protein–protein interaction domain is referred to as Duf38 (Pfam in [35]) or FOG-2 homology domain (FTH) [25]. F-box proteins are often core components of the Skp1/Cullin/F-box-type E3 ubiquitin ligase complexes, and they serve to link specific substrates to the ubiquitin ligase machinery for subsequent proteolysis [36]. However, FOG-2 cannot target GLD-1 for degradation since both function to promote hermaphrodite spermatogenesis [25] (Figure 1A). Current data suggest that the formation of a FOG-2/GLD-1/*tra-2* mRNA ternary complex mediates translational repression of *tra-2* and a corresponding reduction in feminizing activity to allow hermaphrodite spermatogenesis [24,25] (Figure 1B).

The completion of the C. elegans genome sequence [4] and the 10X sequence (representing more than 98% coverage) of the closely related species C. briggsae [5] permits studies of the evolution of sex determination and the inception of hermaphrodite spermatogenesis in morphologically comparable species. Here, we pose the question, do C. elegans and C. briggsae specify male sexual fate in the hermaphrodite germline similarly?

We find that 30 of 31 C. elegans sex determination genes have C. briggsae orthologs, indicating that there is extensive conservation of sex determination pathway components; the lone exception is *fog-2*. We provide evidence that the essential role of FOG-2 in C. elegans hermaphrodite spermatogenesis evolved from post-speciation duplication and divergence of the *fog-2*-related (FTR) gene family and that a *fog-2* gene is not present in C. briggsae. Furthermore, double-stranded-RNA-mediated interference (RNAi) of the *gld-1* ortholog in C. briggsae results in masculinization of the germline instead of the feminization of the germline phenotype observed in C. elegans. The lack of a potential *C. briggsae fog-2* combined with the opposite sex determination function of GLD-1 in C. briggsae indicate that the control of hermaphrodite spermatogenesis, while using most of the same gene products, is fundamentally different between the species and is likely to have evolved independently.

## Results

### Components of Sex Determination Pathway Are Conserved between C. elegans and C. briggsae

To survey conservation in the sex determination pathway between C. elegans and C. briggsae we used reciprocal best BLAST [37,38,39] to identify potential C. briggsae orthologs of 31 known C. elegans sex determination genes, some of which have been previously identified. The 31 genes included 16 that function only in germline sex determination, seven that function in both somatic and germline sex determination, two that function only in somatic sex determination, and six that coordinate sex determination and dosage compensation. We found that 30 of 31 genes have C. elegans–to–C. briggsae reciprocal best BLAST hits and alignments consistent with a high level of conservation (Table 1). Using this method, putative orthologs of all known sex determination genes, including less conserved members, and previously identified genes were recovered [17,26,40,41,42,43,44], with the notable exception of *fog-2*.

**Table 1:**
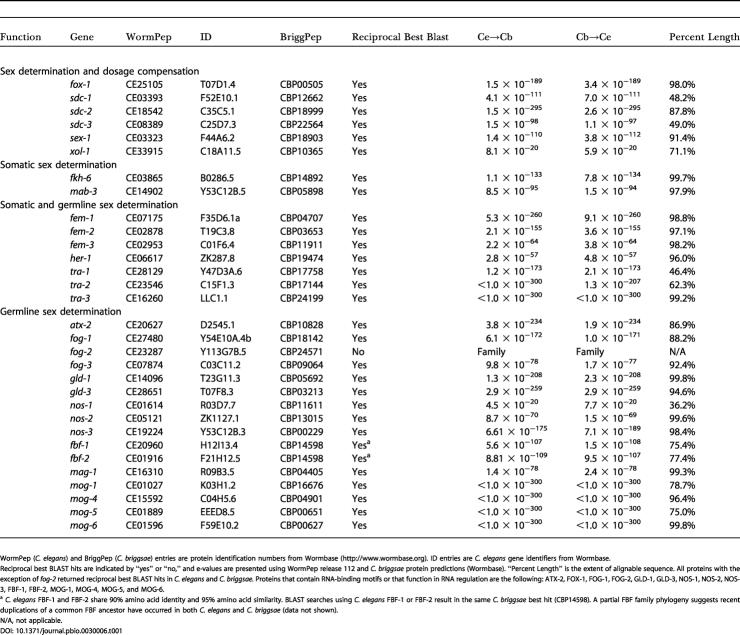
Comparative Analysis of Sex Determination Genes in C. elegans and C. briggsae

WormPep (*C. elegans*) and BriggPep (*C. briggsae*) entries are protein identification numbers from Wormbase (http://www.wormbase.org). ID entries are C. elegans gene identifiers from Wormbase

Reciprocal best BLAST hits are indicated by “yes” or “no,” and e-values are presented using WormPep release 112 and C. briggsae protein predictions (Wormbase). “Percent Length” is the extent of alignable sequence. All proteins with the exception of *fog-2* returned reciprocal best BLAST hits in C. elegans and *C. briggsae.* Proteins that contain RNA-binding motifs or that function in RNA regulation are the following: ATX-2, FOX-1, FOG-1, FOG-2, GLD-1, GLD-3, NOS-1, NOS-2, NOS-3, FBF-1, FBF-2, MOG-1, MOG-4, MOG-5, and MOG-6

^a^ C. elegans FBF-1 and FBF-2 share 90% amino acid identity and 95% amino acid similarity. BLAST searches using C. elegans FBF-1 or FBF-2 result in the same C. briggsae best hit (CBP14598). A partial FBF family phylogeny suggests recent duplications of a common FBF ancestor have occurred in both C. elegans and C. briggsae (data not shown)

N/A, not applicable

The functions of seven C. briggsae sex determination genes have been tested, and current data indicate that these genes exhibit similar and possibly identical functions in C. elegans and C. briggsae (*her-1* [43], *tra-2* [21], *fem-1* [A. Spence, personal communication], *fem-2* [45], *fem-3* [41], *fog-3* [42], and *tra-1* [17]). Importantly, the epistatic relationship and function of two key regulators of sex determination, *tra-2* and *fem-3,* are essentially intact between the sister species in somatic sex determination [21,41] (Figure 1A). At first glance, given the conservation of 30/31 sex determination genes, similar or identical functions for 7/7 genes tested, and maintenance of a key epistatic relationship, it would appear that the sex determination pathway is generally conserved between C. elegans and C. briggsae. However, genetic and molecular studies will be required to determine whether the C. briggsae orthologs are functionally equivalent to their C. elegans counterparts.

A single FOG-2 ortholog could not be resolved by reciprocal best BLAST or by using the reciprocal smallest distance algorithm [46], which uses global sequence alignment and maximum likelihood estimation of evolutionary distances, to infer putative orthologs (data not shown). This indicates that *fog-2* is either highly diverged, present in an unsequenced portion (<2%) of the C. briggsae genome, or potentially a C. elegans–specific adaptation not present in C. briggsae.

### *fog-2* Is a C. elegans–Specific Adaptation

FOG-2 is part of a large, highly diverged F-box- and DUF38/FTH-containing protein family in C. elegans with more than 100 members referred to as FTR proteins [25,36]. The FTR family is also expanded in *C. briggsae,* making the identification of a single functionally equivalent ortholog from a large number of paralogs difficult. Therefore, to discern the relationships among C. elegans and C. briggsae FTR family members, 30 C. elegans and C. briggsae FTR proteins or protein predictions closely related to FOG-2 were used to generate a neighbor-joining phylogeny. The remaining, more diverged FTR members from either species were not included in the phylogeny to avoid long branch attraction [47].

**Figure 2:**
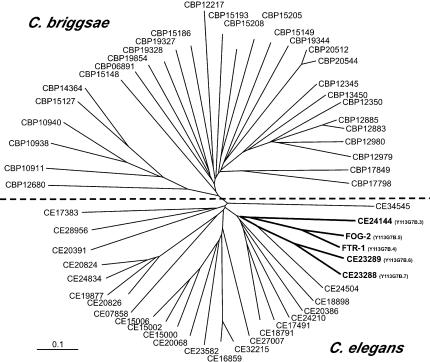
The FTR Gene Family in C. elegans and C. briggsae A radial phylogram showing the relationships of 30 C. elegans and C. briggsae FTR genes closely related to FOG-2 was generated using neighbor-joining. C. elegans and C. briggsae protein predictions with complete F-box and Duf38/FTH (FTR proteins) were identified using BLAST and HMMs, aligned using CLUSTALW, trimmed, de-gapped, and realigned (see Materials and Methods). A clear separation of C. elegans (below dashed line) and C. briggsae (above dashed line) FTR proteins is indicated by the phylogeny. The branch containing FOG-2 and FTR-1 is in bold. Tree is unrooted, and branch lengths are proportional to divergence (also see Figure S1). Bar represents 0.1 substitutions per site. FOG-2 and FTR-1, across their entire length, are more similar to each other than to any other gene in C. elegans. Comparison of the diverged approximately 40aa C-terminal region from both proteins to the closely related FTR genes in the FOG-2 cluster reveals 48% average pairwise identity between these FTRs and FTR-1 and 22% average pairwise identify between these FTRs and FOG-2 (Figure S2). One interpretation of this greater similarity is that FTR-1 may be ancestral; however, it is not clear whether the slight increase in similarity over about 40aa is significant or whether selection rather than evolutionary history produced the sequence similarity observed.

The C. elegans and C. briggsae FTR phylogeny reveals that all of the C. elegans FOG-2 relatives form a single clade and all of the C. briggsae relatives a distinct clade. An unrooted radial phylogram illustrating C. elegans and C. briggsae FTR relationships is presented in Figure 2, and a rectangular representation of the same phylogeny with bootstrap support information is shown in Figure S1. If a closely related homolog of C. elegans FOG-2 were present in C. briggsae the expectation is that it would have clustered with the C. elegans proteins. Contrary to this, the phylogenetic separation of C. elegans and C. briggsae FTR family members into distinct lineages indicates that extensive expansion in the FTR family occurred post-speciation and that C. elegans and C. briggsae FTR genes do not have one-to-one orthologous relationships.

The above results could be misleading if a closely related *C. briggsae fog-2* homolog were present in the less than 2% of the genome sequence that is not present in the final assembly or if the *fog-2* ortholog diverged sufficiently such that the computational methods were not able to distinguish between orthologous and paralogous relationships. To address these possibilities we used low-stringency cross-species Southern blotting in an effort to identify closely related *fog-2*-like sequences in unsequenced portions of the C. briggsae genome, and we used conserved synteny in an attempt to identify a diverged *fog-2* ortholog that might reside in the same genomic location. Both approaches were used to effectively identify other diverged sex determination genes from *C. briggsae* (*tra-2, her-1,* and *fem-2*) prior to the release of the C. briggsae genome sequence [40,43,44].

**Figure 3:**
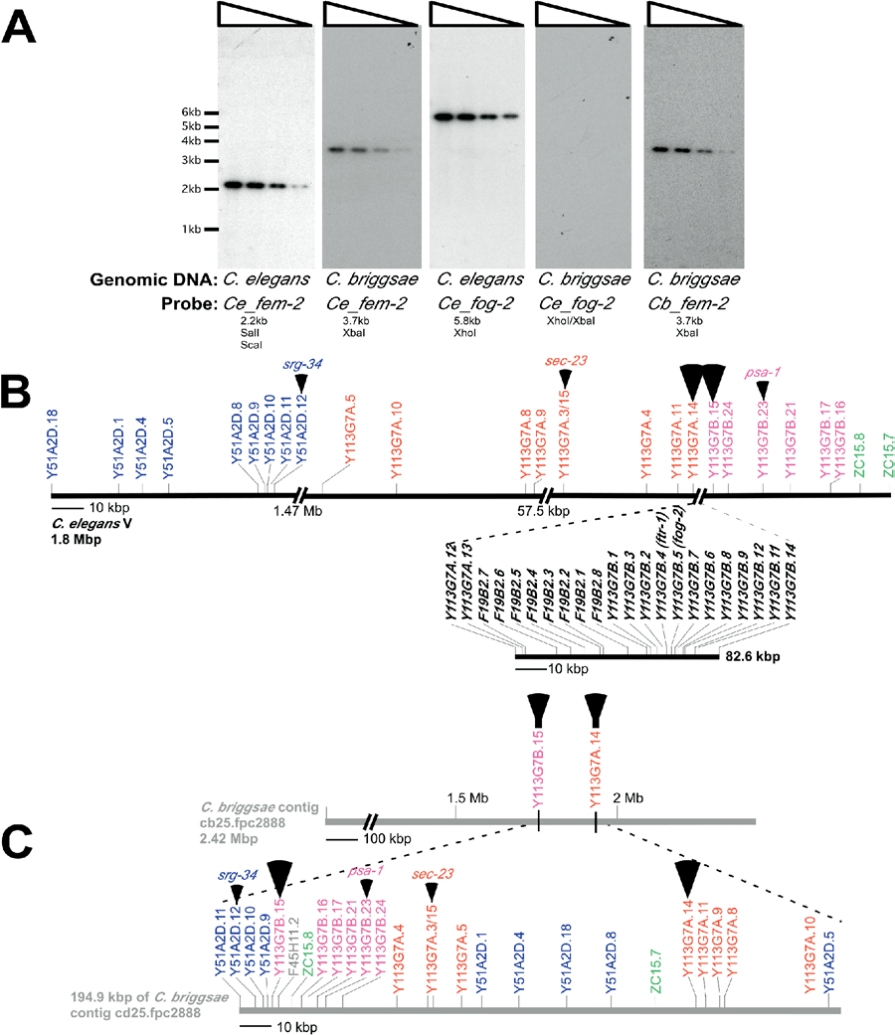
*fog-2* Is Likely Absent in C. briggsae Low-stringency Southern blotting (A) and conservation of synteny (B and C) were used in an attempt to identify a potential *fog-2* gene in C. briggsae. (A) A total of 2–20 ug of digested genomic DNA was used in low-stringency Southern blotting. *C. elegans fem-2* probe (*Ce*_*fem-2*) was able to detect *fem-2* on both same-species and cross-species blots (first two panels). The *C. elegans fog-2* probe (*Ce_fog-2*), which detects both *fog-2* and *ftr-1* on the 5.8-kb XhoI fragment, produced a signal with C. elegans but not C. briggsae genomic DNA (next two panels). *fog-2* cross-species blot integrity was verified by stripping and reprobing with same-species *C. briggsae fem-2* (final panel). Same-species exposures were 4 h and cross-species were 4 d. The *C. elegans fem-2* probe is 70% identical to the C. briggsae genomic sequence. (B) Scale diagram of the C. elegans Chromosome 5 region containing *fog-2*. A 82.6-kb enlargement below, indicated by the dashed lines, shows the *fog-2* cluster containing five canonical FTR genes, one FTR gene with divergent structure, and 16 non-FTR genes (also see Table S1). (C) C. briggsae contig from the genome assembly containing flanking regions with conserved synteny. A 194.4-kb enlargement below, indicated by the dashed lines, covers the C. briggsae region that is predicted to contain a putative *fog-2* ortholog. The conserved genes used to identify the C. briggsae contig are indicated by the arrowheads, with the genes flanking *fog-2* indicated by the large arrowheads. Each gene from the C. briggsae contig with an ortholog defined as a reciprocal best BLAST hit is present on both maps (B and C), and blocks of synteny defined by the C. elegans organization are in the same color. Only one (Y113G7B.11) of the 22 genes from the 82.6-kb *fog-2* cluster was found to have a reciprocal best BLAST hit in C. briggsae (contig cb25.fpc0129, corresponding to the predicted gene CBG05618; Table S1). No FTR genes or genes related to those in the *fog-2* cluster were found within 50-kb on either side of *CBG05618,* indicating that this region does not share conserved synteny with the *fog-2* cluster. Instead, the potential C. briggsae ortholog of Y113G7B.11 is located on a C. briggsae contig region that shows extensive conserved synteny with a different portion of C. elegans Chromosome 5 not involving the *fog-2* cluster (Table S2).

For low-stringency Southern blotting we used a *C. elegans fog-2* probe and a *fem-2* positive control probe against C. briggsae genomic DNA. Under conditions that detected cross-species hybridization with the *C. elegans fem-2* probe against C. briggsae genomic DNA [40], no C. briggsae signal was observed with the *C. elegans fog-2* probe (Figure 3A). This suggests either that a close *fog-2* relative is not present in the less than 2% of the C. briggsae genome that is unsequenced or that it has diverged significantly beyond the level of *fem-2*.

For analysis of conserved syntenic relationships, five conserved C. elegans genes surrounding *fog-2* (*srg-34, sec-23, psa-1,* Y113G7A.14, and Y113G7B.15) were used to query C. briggsae contigs. The genes *srg-34, sec-23,* and *psa-1* are highly conserved across metazoans and have reciprocal best BLAST hits in C. briggsae (Figure 3B and 3C, small arrow heads). The genes Y113G7A.14 and Y113G7B.15 flank the gene-dense *C. elegans fog-2* region and also have reciprocal best BLAST hits in C. briggsae (Figure 3B and 3C, large arrow heads). All five genes were found to be represented on a single C. briggsae contig, suggesting that the global syntenic relationships are conserved, but with detailed analysis revealing a number of differences in gene order (Figure 3B and 3C). However, *fog-2*, its four adjacent close FTR relatives, and 16 surrounding genes in an 82.6-kb region were absent from this C. briggsae contig, while the conserved genes on either side were present (Table S1 and S2).

The closest relative of *fog-2* is the gene *ftr-1,* which is part of a group of five closely related *ftr* genes that are colinear in C. elegans and not present in C. briggsae [25] (Figures 2 and 3). If *fog-2* and *ftr-1* are the result of a “recent” post-speciation duplication within the C. elegans lineage, as suggested by the phylogeny, then we would expect that fewer synonymous substitutions (K_s_) have occurred between *fog-2* and *ftr-1* relative to other *C. elegans/C. briggsae* best BLAST orthologs. Consistent with a recent duplication, the K_s_ for *fog-2/ftr-1* is not saturated (K_s_ = 0.36) whereas the average K_s_ for reciprocal best BLAST hits between C. elegans and C. briggse is saturated (K_s_ = 1.72) [5].

The finding that *fog-2* and *ftr-1* arose from a relatively recent local duplication within C. elegans strongly supports the contention that *fog-2* is not present in C. briggsae. These results imply that C. briggsae must regulate hermaphrodite spermatogenesis differently than C. elegans.

### The Diverged C-Terminal of FOG-2 Is Necessary for GLD-1 Binding

**Figure 4:**
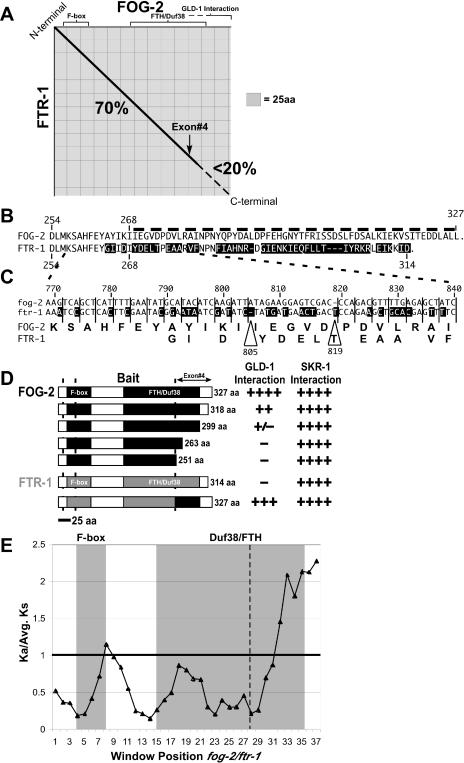
The Highly Diverged FOG-2 C-Terminal Region Is Responsible for GLD-1 Interaction in C. elegans (A) Dot plot of FOG-2/FTR-1, with the black diagonal line delimiting regions of greater than 70% identity based on a 10-aa sliding window. The dashed horizontal line at the C-terminus indicates a region of low identity. The arrow indicates the final exon 4 boundary. (B) Protein sequence alignment of FOG-2 and FTR-1 encoded by exon 4. Differences are shaded in black and illustrate the abrupt breakdown in sequence conservation. The dashed line marks the region required for GLD-1 interaction. (C) Nucleotide alignment of *fog-2* and *ftr-1* EST coding regions expanded from a portion of the protein sequence alignment, with vertical lines delimiting the reading frame relative to *fog-2.* Amino acid sequence for FOG-2 (above) and changes in FTR-1 (below) are below the alignment. Frame-shifting indels are indicated by the large open arrowheads. (D) The C-terminal FOG-2 region is required for GLD-1 interaction in the yeast two-hybrid system. Full-length FOG-2 (black) and FTR-1 (grey) constructs were tested for interaction with GLD-1. FOG-2 interacts with GLD-1 (++++) whereas FTR-1 does not (−). Progressive C-terminal deletions (black) in FOG-2 were generated to identify FOG-2 requirements for GLD-1 interaction. Binding to GLD-1 was completely eliminated with the removal of the C-terminal 64 aa of FOG-2 exon 4. Transfer of exon 4 to FTR-1 (grey/black chimera) resulted in the transfer of GLD-1 binding to FTR-1. Control interactions to test for the production of functional proteins were performed with the Skp1 homolog SKR-1, which binds to the F-box region (see Materials and Methods). Searches for C. elegans and C. briggsae proteins with homology to the 64-aa FOG-2 region required for GLD-1 interaction (or FOG-2 exon 4) failed to identify any predicted proteins with significant homology (>35% or e-value = 0.01) other than FTR-1, which cannot bind GLD-1 and does not compensate for FOG-2 in sex determination. (E) Sliding-window (100-nt window, 25-nt shift) estimation of K_a_/K_s_ ratio for *fog-2/ftr-1* using full-length average K_s_. The K_a_/K_s_ ratio is highest at the C-terminal end of the Duf38/FTH domain, reaching a peak of 2.2 in window 37. The position of the F-box and Duf38/FTH domain are indicated by grey shading. The bold horizontal line is at the K_a_/K_s_ = 1 threshold. The dashed vertical line indicates the boundary between exon 3 and exon 4.

Previous work has shown that FOG-2 is an integral part of the *tra-2* 3′ UTR translational repression complex. The RNA-binding protein GLD-1 makes direct contact with the *tra-2* 3′ UTR, and FOG-2 is recruited to the complex via its interaction with GLD-1 [24,25]. In spite of the high similarity between *fog-2* and *ftr-1* (Figure 4), *ftr-1* cannot compensate for *fog-2* in the promotion of hermaphrodite spermatogenesis [25]. This indicates that *fog-2* must contain unique sequences that allow it to function in sex determination.

Pairwise comparisons between FOG-2 and FTR-1 reveal a highly diverged C-terminal region encoded by the final exon (exon 4) (Figure 4A–4C). Before the C-terminal region of low similarity, the relative reading frames of *fog-2* and *ftr-1* are conserved with all insertions and deletions in three nucleotide multiples and an overall amino acid identity of 70%. Within the final exon, multiple amino acid substitutions, insertions, and deletions have occurred, resulting in a region of low nucleotide and amino acid identity (Figure 4B and 4C). For example, an indel (deletion relative to *fog-2*) at nucleotide 805 shifts the reading frame of FOG-2 relative to FTR-1 and results in a region of low similarity between the proteins (Figure 4B). A second indel at position 819 restores the reading frame but additional substitutions result in a diverged amino acid sequence (Figure 4C).

The dramatic differences between the FOG-2 and FTR-1 C-terminal regions suggested a connection between the unique functionality of FOG-2 in sex determination and the highly diverged C-terminal region. Since FOG-2 interacts with GLD-1 and both are required for the promotion of the male germ cell fate in the hermaphrodite, we determined whether the diverged FOG-2 C-terminal region was necessary for its interaction with GLD-1 (Figure 4). Progressive C-terminal deletions of FOG-2 were tested for their ability to interact with GLD-1 in the yeast two-hybrid system (Figure 4D). Full-length FOG-2 interacts with GLD-1 [25]; however, C-terminal deletions of nine and 28 aa in FOG-2 reduced the interaction, and deletion of 64 and 76 aa (essentially all of exon 4) eliminated the interaction (Figure 4D), indicating that the highly divergent C-terminal region is necessary for GLD-1 binding. All full-length and deletion constructs were tested against the Skp1 homolog SKR-1 as a positive control for functionality in the two-hybrid system (see Materials and Methods).

To determine whether the C-terminal region of FOG-2 is sufficient to confer GLD-1 interaction, an FTR-1/FOG-2 exon 4 chimera was generated and assayed for its ability to interact with GLD-1. Normally FTR-1 lacks the ability to interact with GLD-1 [25] (Figure 4D). The replacement of exon 4 from *ftr-1* with exon 4 from *fog-2* allowed the chimera to interact with GLD-1 (Figure 4D). Thus, the C-terminal 74aa region of FOG-2, when in the context of the FTR-1 F-box and Duf38/FTH sequences, is sufficient to confer GLD-1 binding.

### FOG-2/GLD-1 Interaction Evolved Rapidly in C. elegans

Gene duplication provides the raw material for the evolution of novel adaptations, having been implicated in the diversity of the host–pathogen immune response, rapid onset of insecticide resistance, and diversity of vertebrate body plans [48]. Rapidly evolving genes, or portions of genes, under positive selection can be identified by comparison of nucleotide alterations that result in amino acid changes (non-synonymous substitutions [K_a_]) to alterations that do not change the amino acid (K_s_) [49,50]. K_a_/K_s_ ratios that are equal to or less than one are indicative of neutral or purifying selection, where substitutions that change amino acids offer no fitness advantage or result in lowered fitness. In contrast, K_a_/K_s_ ratios greater than one, common in rapidly evolving genes, are indicative of positive selection, where non-synonymous changes offer some fitness advantage and are fixed at a higher rate than synonymous substitutions [51].

To determine the selection acting on the *fog-2/ftr-1* duplication we compared K_a_/K_s_ ratios between *fog-2, ftr-1,* and the five FTR genes closest to *fog-2* in C. elegans. Pairwise comparisons of codon-delimited full-length coding sequences closely related to *fog-2* suggest that purifying selection dominates along the *fog-2* branch, as all comparisons produced K_a_/K_s_ ratios less than one (mean = 0.46). However, while the overall K_a_/K_s_ ratio for *fog-2/ftr-1* is not indicative of positive selection (mean = 0.58), sliding-window K_a_/K_s_ ratio estimates [52] for *fog-2* and *ftr-1* indicate that the highly diverged C-terminal region of FOG-2/FTR-1 contains residues under positive selection (K_a_/K_s_ = 1.98 for nucleotides 777–987, windows 33–37) (Figure 4). An alternate method using maximum likelihood estimation of K_a_/K_s_ (PAML and codeml [53]) confirmed the presence of residues under positive selection within the C-terminal region (see Materials and Methods). Thus, the primary differences between FOG-2 and FTR-1 are localized to the rapidly evolving C-terminus of FOG-2 that is required for GLD-1 binding and is under positive selection.

The yeast two-hybrid data, together with the genetics of *fog-2* [25], indicate that FOG-2 is unique among C. elegans FTR genes in functioning with GLD-1 in germline sex determination. Given the specificity of the FOG-2/GLD-1 interaction in *C. elegans,* phylogenetic analysis of FTR proteins (see Figure 2), and additional experiments (see Figures 3 and 4) that indicate that there are no close relatives of *fog-2* among C. briggsae FTR genes, it is unlikely that any C. briggsae FTR protein functions with C. briggsae GLD-1 in sex determination.

**Figure 5:**
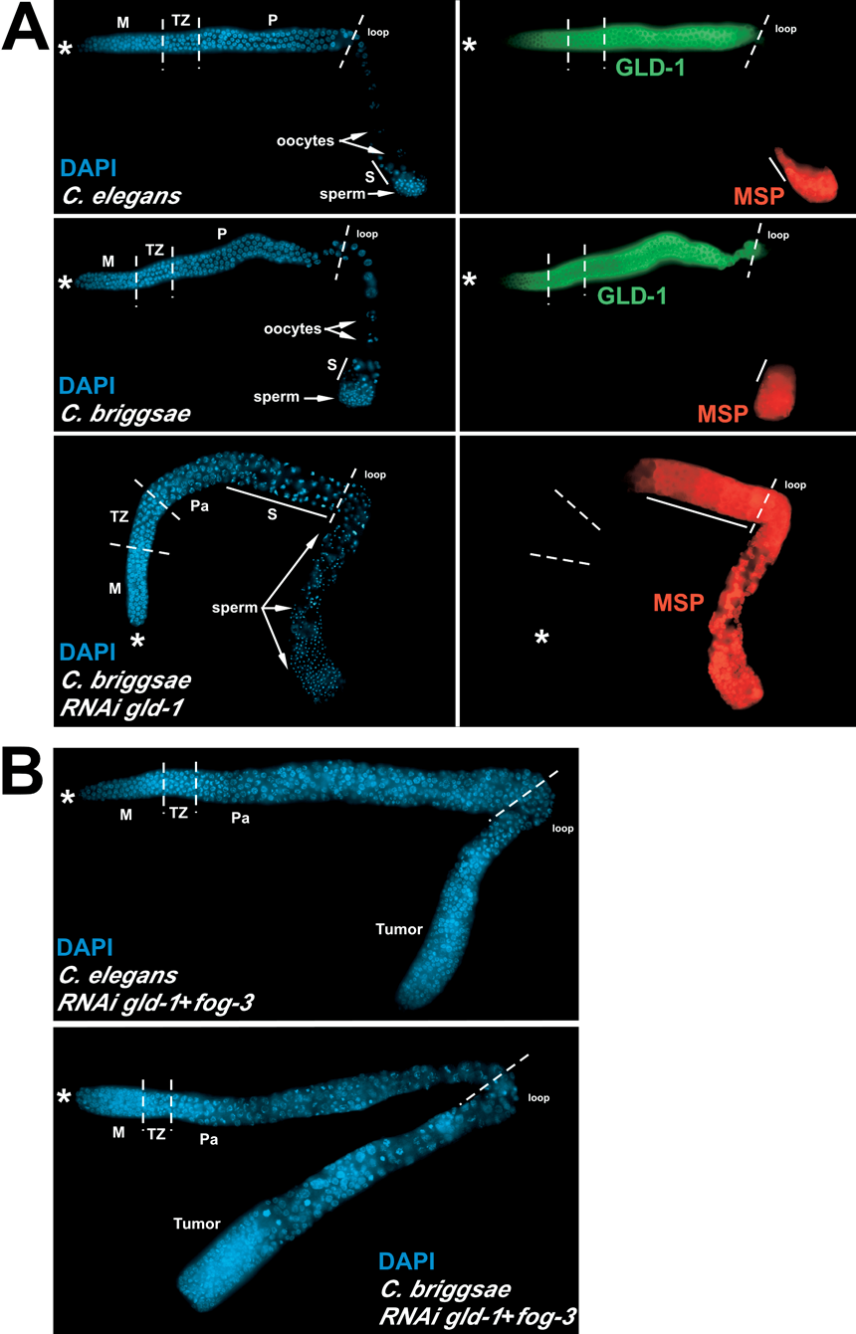
GLD-1 Has the Opposite Sex Determination Function in C. elegans and C. briggsae For (A) and (B) the distal end of the gonad arm is indicated by the asterisk, and regions of the germline are delimited by dashed vertical lines as follows: M, mitotic zone; TZ, transition zone; P, pachytene; Pa, abnormal pachytene; and S, spermatocytes. For both (A) and (B) staining indicated is as follows: DAPI, blue, nuclear DNA; GLD-1, green; and MSP, red. (A) RNAi of *C. briggsae gld-1* results in masculinization of the germline. Paired DAPI-stained (left) and GLD-1- and MSP-stained (right) images of dissected young adult hermphrodite germlines. Top four panels illustrate the similarity between C. elegans and C. briggsae germline morphology and polarity (DAPI, blue; GLD-1, green; MSP, red). In both species, sperm (“sperm” arrow) are produced first before switching to oogenesis (“oocytes” arrow), and the pattern of cytoplasmic GLD-1 accumulation (green) is identical. GFP-injected controls were identical to wild-type animals. *C. briggsae gld-1* RNAi animals exhibit masculinization of the germline (lower panels). A vast excess of sperm extends to the loop region (“sperm” arrows), and spermatogenesis extends further distally (solid line). Masculinization is confirmed by a corresponding extension in MSP staining beyond the loop (compare lower right to controls above). (B) RNAi of *gld-1* and *fog-3* in C. elegans and C. briggsae results in a similar tumorous germline phenotype. C. elegans (top) and C. briggsae (bottom) have normal mitotic, transition, and entry into pachytene, but abnormal progression through pachytene, based on DAPI morphology. Both MSP and GLD-1 staining were below the level of detection in both cases.

In contrast with FOG-2, a highly conserved GLD-1 ortholog is present in C. briggsae (Table 1) and has a germline expression pattern essentially identical to that of C. elegans (Figure 5A, top right and middle right). In fact, C. elegans GLD-1 and C. briggsae GLD-1 share 81% amino acid identity overall and more than 90% in the maxi-KH RNA-binding region. Since FOG-2 and GLD-1 function together to promote the male germ cell fate in C. elegans hermaphrodites, this raised the question of what role, if any, C. briggsae GLD-1 plays in C. briggsae germline sex determination.

### GLD-1 Has Distinct Functions in C. elegans and C. briggsae Germline Sex Determination

To examine C. briggsae GLD-1 function in sex determination we performed RNAi [54] by injecting double-stranded *C. briggsae gld-1* RNA into C. briggsae adult hermaphrodites followed by phenotypic analysis of F1 self progeny (see Materials and Methods). From genetic analysis of *C. elegans gld-1* [28,29] there are two functions relevant to this study. First, C. elegans GLD-1 has an essential function in meiotic prophase progression during oogenesis. In null mutant hermaphrodites oogenic germ cells progress to pachytene and then return to the mitotic cell cycle, giving rise to ectopic proliferation and a germline tumor [28]. For this function C. elegans GLD-1 acts to spatially restrict the translation of multiple target mRNAs during oogenesis. GLD-1 oogenic target mRNAs are repressed during early meiotic prophase, but then are translated during late meiotic prophase following the loss of GLD-1 at the end of pachytene [30,31,55]. Second, C. elegans GLD-1 is necessary for the specification of the male sexual fate in the hermaphrodite germline. This function is most simply revealed as a haplo-insufficient feminization of the hermaphrodite germline [28,29]. *C. elegans gld-1* has no known essential functions in male meiotic prophase progression or in XO male germline sex determination as C. elegans null males are wild-type [28,29].

C. briggsae GLD-1 may still function as a translational repressor of *C. briggsae tra-2* mRNA even in the absence of a FOG-2 ortholog. This is a possibility because FOG-2 is not required for C. elegans GLD-1 binding to the *C. elegans tra-2* mRNA in vitro [25], and some conservation is preserved between the C. elegans and *C. briggsae tra-2* 3′ UTRs [34]. In this case, RNAi of GLD-1 in C. briggsae might feminize the germline given that *C. briggsae tra-2* promotes female development in both the germline and soma [21]. Alternatively, C. briggsae GLD-1 might have no role in germline sex determination, in which case RNAi would not result in a sex determination phenotype.

Surprisingly, *C. briggsae gld-1* RNAi resulted in a masculinized germline (Figure 5A, bottom; Table 2), with no effect on the soma. Staining with 4′,6′-diamidino-2-phenylindole hydrochloride (DAPI) and anti–major sperm protein (MSP) (see Materials and Methods) revealed continuous spermatogenesis leading to a vast excess of sperm at the expense of oogenesis. Anti-GLD-1 antibody staining of *gld-1* RNAi F1 gonad arms indicated that the level of GLD-1 protein was reduced to below detectable limits (Figure 5A, bottom right). C. briggsae control hermaphrodites injected with double-stranded RNA for green fluorescent protein (GFP) had gonad morphology identical to wild-type (Figure 5A, top left and middle left). The masculinized phenotype of *gld-1* RNAi in C. briggsae indicates that the wild-type function of GLD-1 in C. briggsae is to promote the female germ cell fate, likely by the translational repression of an mRNA that encodes a masculinizing gene product. This function is in direct contrast to that of C. elegans GLD-1, which promotes the male germ cell fate by translational repression of the feminizing *tra-2* mRNA.

**Table 2:**
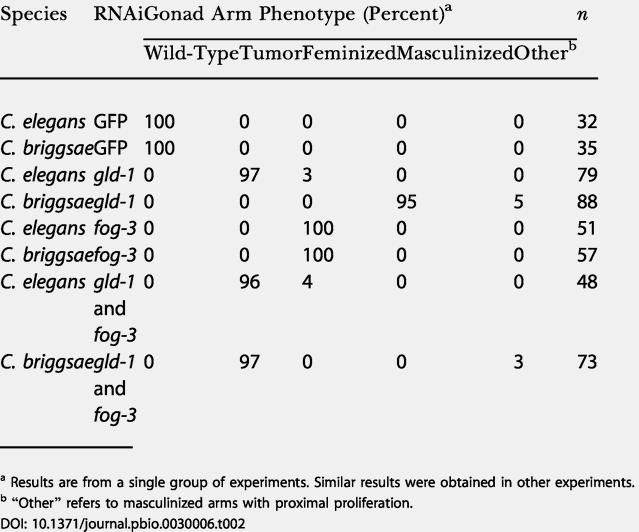
Summary of GLD-1 RNAi Germline Phenotype in C. elegans and C. briggsae

^a^ Results are from a single group of experiments. Similar results were obtained in other experiments

^b^ “Other” refers to masculinized arms with proximal proliferation

### GLD-1 Function in Meiotic Prophase Progression during Oogenesis Is Conserved

Given the difference in sex determination function, it is possible that C. elegans and C. briggsae GLD-1 have few conserved functions in germline development. To investigate this we took advantage of well-defined activities of *gld-1* in C. elegans such as its essential function in female meiotic prophase progression and in the translational repression of the evolutionarily conserved yolk receptor mRNA encoded by the *rme-2* locus [28,31].

The *gld-1*-null tumorous phenotype results from aberrant oogenic prophase progression and a return to mitosis [28,29]. This phenotype is dependent on germline sex because a tumor only occurs when germ cell fate is set to female [28,29]. The masculinized phenotype caused by *gld-1* RNAi in C. briggsae is likely to preclude the detection of this function as the *C. elegans gld-1*-null tumorous phenotype is suppressed by mutations that cause masculinization of the germline [29]. To overcome the masculinization we combined *fog-3* RNAi with *gld-1* RNAi in C. briggsae. Since *C. elegans fog-3* functions near the end of the sex determination pathway and in *C. briggsae fog-3* RNAi results in feminization of the germline [42], we predicted that *C. briggsae fog-3* RNAi would be epistatic to the masculinization of the germline of *C. briggsae gld-1* RNAi.

Similar to the *C. elegans gld-1*-null, RNAi of *gld-1* or *gld-1* and *fog-3* in C. elegans and double RNAi of *gld-1* and *fog-3* in C. briggsae resulted in a robust proximal germline tumor (Figure 5B; Table 2). Control RNAi with *fog-3* alone resulted in feminized germlines in both species [42]. Both the mitotic zone and transition zone appear to have roughly normal nuclear morphology, with more proximal nuclei having abnormal pachytene morphology (Figure 5B), suggesting that germ cells are entering meiosis but progressing aberrantly before returning to mitosis. The return-to-mitosis tumorous phenotype in each species was confirmed using phosphohistone H3 staining, a mitotic proliferation marker [56]. We cannot rule out the possibility that the C. briggsae phenotypes observed, masculinization of the germline with *gld-1* RNAi alone and tumorous germline with *gld-1* and *fog-3* RNAi, are the result of incomplete knockdown leading to partial *gld-1* loss of function.

The *rme-2* yolk receptor mRNA is a known target of GLD-1-mediated translational repression in C. elegans [31]. In *C. elegans,* GLD-1 and RME-2 have mutually exclusive expression patterns because *rme-2* mRNA is translationally repressed in the transition zone and pachytene region, where GLD-1 levels are high, and translated in oocytes, where GLD-1 levels are low [31]. In *C. elegans gld-1*-null germlines RME-2 is ectopically expressed in the transition zone and pachytene region owing to loss of GLD-1-mediated translational repression of the *rme-2* mRNA [31].

**Figure 6:**
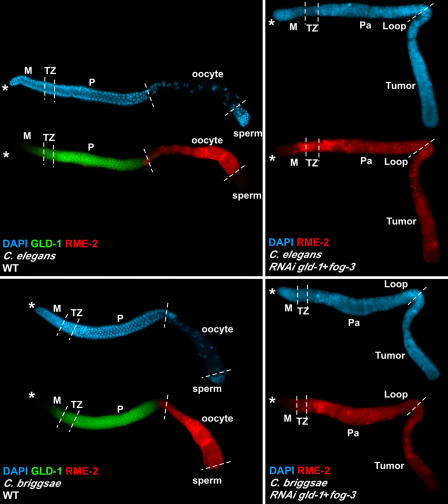
GLD-1-Mediated Translational Repression of *rme-2* mRNA in C. elegans and C. briggsae In both C. elegans and C. briggsae wild-type (WT) animals (left panels), GLD-1 (green) and RME-2 (red) have mutually exclusive accumulation patterns. In C. elegans (upper right), *gld-1* and *fog-3* RNAi results in a germline tumor with ectopic RME-2 accumulation (red expanded). In C. briggsae (lower right), RNAi of *gld-1* and *fog-3* also results in germline tumor with ectopic RME-2 accumulation (red expanded). The germline tumor and expansion of RME-2 expression due to ectopic translation are similar between the two species (compare right top and bottom, DAPI [blue]). The distal end of the gonad arm is indicated by the asterisk, and regions of the germline are delimited by dashed vertical lines. DAPI, blue, nuclear DNA; GLD-1, green; RME-2, red; M, mitotic zone; TZ, transition zone; P, pachytene; Pa, abnormal pachytene.

A similar, mutually exclusive accumulation pattern in C. briggsae suggests that C. briggsae GLD-1 is a translational repressor of *C. briggsae rme-2* mRNA (Figure 6). To determine whether C. briggsae GLD-1 represses the *rme-2* mRNA, double RNAi of *gld-1* and *fog-3* was performed in both species, and gonad arms were stained for RME-2 protein [57]. Reduction of GLD-1 and FOG-3 by RNAi results in the ectopic accumulation of RME-2 protein in both C. elegans and C. briggsae (Figure 6), indicating that the role of GLD-1 in the translational repression of the *rme-2* mRNA is conserved. Thus, despite the opposite roles of GLD-1 in sex determination, essential functions of GLD-1 in oogenesis are conserved between the species.

## Discussion

Our results indicate that the control of hermaphrodite spermatogenesis is fundamentally different between the sister species C. elegans and C. briggsae at the level of FOG-2/GLD-1/*tra-2* mRNA regulation. While FOG-2 is essential for self-fertile hermaphroditism in *C. elegans,* a closely related homolog of FOG-2 could not be recovered in C. briggsae by reciprocal best BLAST, phylogenetic inference, low-stringency hybridization, or analysis of conserved synteny*.* Comparison of synonymous changes between *fog-2* and its closest relative, *ftr-1,* indicates that *fog-2* is the product of a recent expansion “specific” to C. elegans in the FTR gene family and implies that the evolution of FOG-2 and its incorporation into the sex determination pathway occurred post-speciation. Consistent with this, the C-terminal region of FOG-2 required for binding to GLD-1 was found to be highly diverged and “unique” to FOG-2 in C. elegans. Interestingly, GLD-1 was found to have a sex determination function in C. briggsae opposite that in C. elegans while retaining similar functions in female meiotic prophase progression and oogenesis. The absence of FOG-2, and the opposite sex determination function of GLD-1, provides evidence for the independent evolution of hermaphroditism in C. elegans and C. briggsae.

### General Conservation of the Sex Determination Pathway

Reciprocal best BLAST indicates that C. elegans and C. briggsae have orthologs of 30 of 31 known sex determination pathway genes. Conserved functions for *C. briggsae her-1, tra-2, fem-1, fem-2, fem-3, fog-3,* and *tra-1* have been demonstrated by transgene rescue of C. elegans mutations or similarity of RNAi loss-of-function phenotype [17,21,26,41,42,43,45]. The general conservation of genes that govern sex determination suggests that the underlying pathway remains largely intact between the species.

RNAi and transgenic experiments have suggested that while *fem-2* and *fem-3* have conserved roles in the somatic sex determination of both species, they may play diminished roles in C. briggsae germline sex determination [41,45]. There are two possibilities that could explain these results. One is that there are inherent species-specific differences in susceptibility to RNAi or in the ability to reconstitute complete gene function by transgene rescue. The other is that differences in C. elegans and C. briggsae phenotypes reveal functional divergence in sex determination pathway components. Analysis of null mutations in C. briggsae orthologs of C. elegans sex determination genes will help to distinguish between these possibilities. While some functional differences may turn out to be valid, *tra-2* (feminizing) and *fem-3* (masculinizing) apparently play the same somatic roles in both species, and their epistatic relationship appears to be conserved [41].

### *fog-2* Is Unique to C. elegans

Within the context of general conservation of sex determination pathway components and conserved key epistatic relationships, the absence of *fog-2* in C. briggsae is intriguing. *fog-2* arose as a consequence of recent C. elegans–specific gene duplication events, and none of the closely related *C. elegans fog-2* paralogs can compensate for loss of *fog-2* in sex determination [25]. Thus, it is unlikely that more distantly related C. briggsae FTRs are involved in GLD-1/*tra-2*-mRNA-mediated promotion of hermaphrodite spermatogenesis. Since *fog-2* is essential for the promotion of spermatogenesis in C. elegans hermaphrodites and is not present in *C. briggsae,* the direct implication is that specification of the male germ cell fate in C. briggsae hermaphrodites is fundamentally different from that in C. elegans and that it evolved independently.

The highly diverged C-terminus of FOG-2 is under positive selection and is necessary and sufficient for GLD-1 binding within the context of an F-box and FTH domain (see Figure 4). Acquiring the diverged C-terminus was crucial in FOG-2 becoming incorporated into the sex determination pathway. With respect to the C. elegans lineage, it is unclear whether *fog-2* retains an ancestral function in sex determination and *ftr-1* has changed/drifted away or, alternatively, whether *ftr-1* represents the ancestral function and *fog-2* has recently evolved a role in sex determination (also see Figure S2). The *ftr-1* gene is expressed, though its function is currently unknown. RNAi of *ftr-1* into the *fog-2* null did not reveal any obvious phenotypes beyond feminization of the germline [25].

### Conserved GLD-1 Functions in C. elegans and C. briggsae Meiotic Prophase during Oogenesis

GLD-1 function in meiotic prophase progression and oogenesis shows substantial conservation between the species (see Figures 5 and 6), which is not surprising given the high level of sequence conservation between C. elegans and C. briggsae GLD-1. This is illustrated by the *rme-2* yolk receptor mRNA being regulated similarly between the species (Figure 6). Current data indicate that C. elegans GLD-1 binds to, and likely represses translation of, more than 100 mRNA targets [31,55] (M.-H. Lee, V. Reinke, and T. Schedl, unpublished data). The *C. elegans gld-1*-null tumorous phenotype likely results from misregulation of multiple mRNA targets [31]. While the identity of the misregulated mRNA targets causing the *gld-1*-null tumorous phenotype are currently unknown, the fact that *C. briggsae gld-1* and *fog-3* RNAi results in a similar tumorous phenotype suggests that a similar, if not identical, set of C. briggsae GLD-1 mRNA targets are misregulated. The absence of a FOG-2 ortholog in C. briggsae is unlikely to have a major effect on GLD-1-mediated translational control since FOG-2 appears to be required only as a cofactor for *tra-2* repression [25,27,31,55,58]. Thus, it is possible that the majority of GLD-1 mRNA targets involved in prophase progression and oogenesis are regulated similarly between species.

### Divergent GLD-1 Function in C. elegans and C. briggsae Sex Determination

Genetic analysis reveals that C. elegans and C. briggsae GLD-1 have opposite functions in germline sex determination; C. elegans GLD-1 promotes spermatogenesis while C. briggsae GLD-1 promotes oogenesis. This indicates that the major sex determination function of C. briggsae GLD-1 is not translational repression of *tra-2* feminizing activity. C. elegans GLD-1 binds two 28 nucleotide direct repeat elements on the *C. elegans tra-2* mRNA 3′ UTR to mediate translational repression [24]. Somatic reporter gene assays in C. elegans and C. briggsae have suggested that the *tra-2* 3′ UTRs of both species are able to function in translational repression [34], with the implication being that the C. elegans and C. briggsae 3′ UTRs are regulated similarly. However, these data are difficult to interpret in the context of germline sex determination, as GLD-1 and FOG-2 are not natively expressed in the soma and neither GLD-1 nor FOG-2 have essential functions in somatic sex determination [25,27,28,29,30].

One hypothesis to explain our results is that C. briggsae GLD-1 binds to the *C. briggsae tra-2* mRNA but is necessary for translational activation instead of translational repression as in C. elegans. However, for all characterized C. elegans GLD-1 targets, and *C. briggsae rme-2* mRNA, GLD-1 acts as a translational repressor [2,31,55,58,59]. We currently do not understand how FOG-2 acts with GLD-1 in translational repression of *C. elegans tra-2* mRNA. In *C. elegans,* GLD-1 can bind the *tra-2* mRNA in the absence of *fog-2* in worm extracts but cannot properly repress its translation in vivo [25]. This suggests that the role of FOG-2 may be to recruit additional factors specific to the *C. elegans tra-2* mRNA 3′ UTR that allow for efficient GLD-1 translational repression. Assuming C. briggsae GLD-1 binds *C. briggsae tra-2* mRNA in vivo, given the absence of a FOG-2 ortholog, there may be no regulatory consequence of this binding.

Another possibility is that C. briggsae GLD-1 binds and translationally represses an mRNA that promotes spermatogenesis. This could occur if a masculinizing sex determination gene, either present in both species or unique to *C. briggsae,* has come under GLD-1 control in C. briggsae. Given the conservation of GLD-1 and its regulation of at least some common targets (e.g., *rme-2*) it is unlikely that changes in GLD-1 are responsible for a new mRNA target in C. briggsae. Instead, it is more likely that one or more new target mRNAs have acquired sequences that direct GLD-1 binding and translational repression. The requirements for GLD-1 binding are only just being elucidated, with a hexanucleotide sequence being one important feature amid otherwise diverse GLD-1 binding regions [32,55]. Thus, small numbers of changes in UTRs are likely to be sufficient for new mRNAs to come under GLD-1-mediated regulation.

### Evolution of Self-Fertile Hermaphroditism

Current phylogenetic data suggest that hermaphroditism evolved independently in *Caenorhabditis* and other lineages of Rhabditid nematodes from an ancestral female/male state [5,6,7,10,11,60]. This is consistent with our results showing that control of hermaphrodite spermatogenesis at the level of FOG-2/GLD-1/*tra-2* mRNA is fundamentally different between C. elegans and C. briggsae. This raises the question, how might the transition from the ancestral female/male to hermaphrodite/male system of reproduction have occurred multiple times within the *Caenorhabditis* clade?

The anatomy and reproductive physiology of C. elegans allow both sperm that is introduced by mating and sperm that develops within the female gonad of the hermaphrodite to be effectively used in reproduction [14,61,62]. Either source of sperm generates a MSP-derived signal that is required for full-grown oocytes to undergo meiotic maturation, ovulation, and fertilization in the spermatheca [62,63]. Not only is the anatomy conserved but an MSP-derived sperm signal also appears to be utilized by both C. briggsae and C. remanei (a female/male species) to induce oocyte maturation and ovulation [63,64]. This conservation within *Caenorhabditis* indicates that major changes in anatomy and reproductive physiology are not necessary in the transition from female/male to hermaphrodite/male reproduction.

The relative ease with which mutants and mutant combinations can alter the sex determination system in C. elegans has suggested that transitions between mating systems may not be difficult and that the overall sex determination pathway reflects selection for a particular mating system rather than a constant regulatory mechanism [65]. The hermaphrodite pattern of spermatogenesis first then oogenesis is achieved by high masculinizing/low feminizing activity in early larvae followed by low masculinizing/high feminizing activity in late larvae/adults (see Figure 1; reviewed in [18,19,26,66]). Lowering or eliminating germline masculinizing activity in XX animals can convert C. elegans from hermaphrodite/male to female/male reproduction (Table 3, and references therein [20,27,28,29,66,67,68,69]). For example, *fog-2*-null mutations result in strains that reproduce as XX females and XO males. The mutant female/male strains can be converted back to hermaphrodite reproduction by introducing masculinizing mutations in certain genes (e.g., *fog-2*-null; *fem-3-*gf; Table 3). The generality of high masculinizing/low feminizing activity early followed by low masculinizing/high feminizing activity late is borne out by other sets of mutually suppressing feminizing-plus-masculinizing combinations in which the double mutants are self-fertile while each single mutant is usually self-sterile (e.g., *tra-1-*gf; *fem-3*-gf; Table 3). Thus, multiple genetic states can yield self-fertile hermaphrodite/male and male/female reproduction in C. elegans.

**Table 3:**
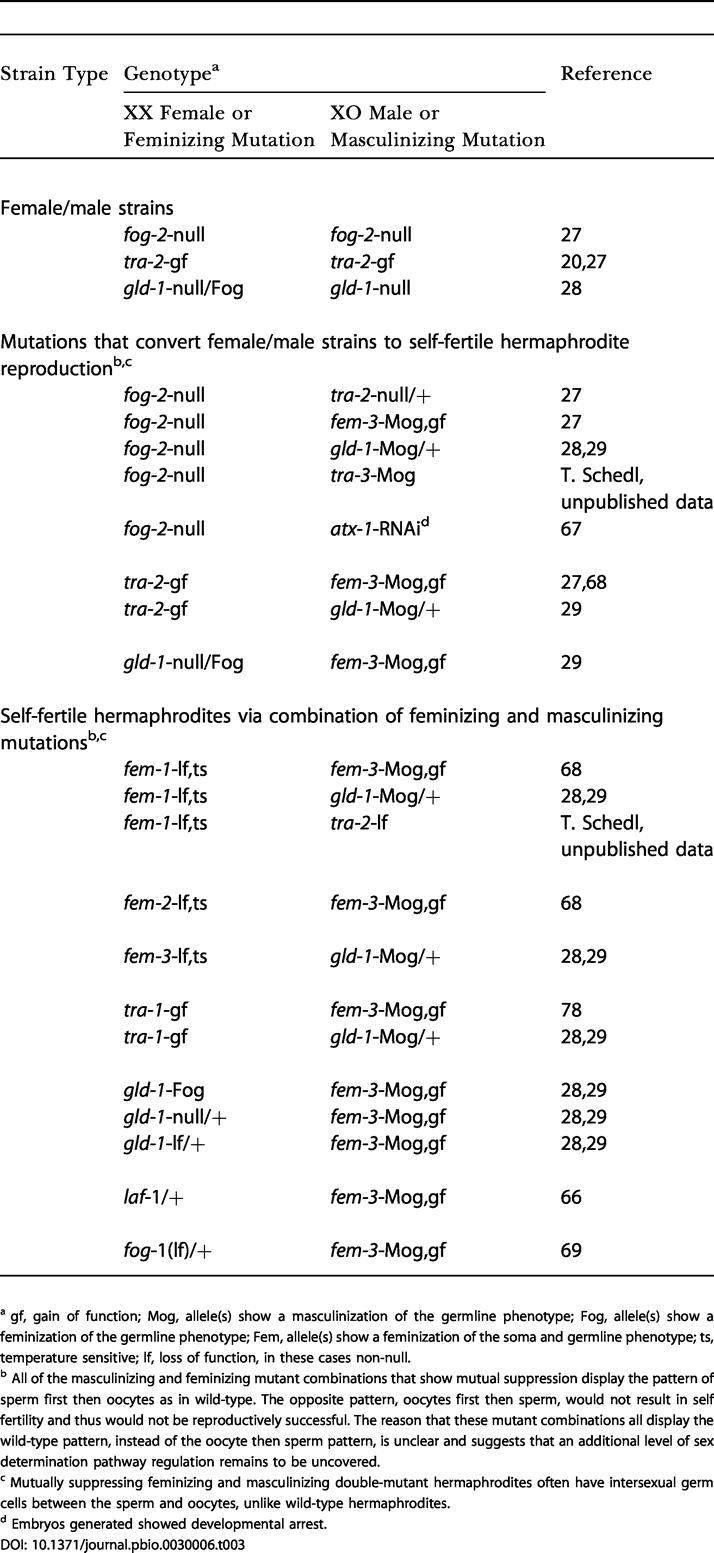
C. elegans Sex Determination Mutants That Yield Female/Male Reproduction and Mutually Suppressed Hermaphrodite Reproduction

^a^ gf, gain of function; Mog, allele(s) show a masculinization of the germline phenotype; Fog, allele(s) show a feminization of the germline phenotype; Fem, allele(s) show a feminization of the soma and germline phenotype; ts, temperature sensitive; lf, loss of function, in these cases non-null

^b^ All of the masculinizing and feminizing mutant combinations that show mutual suppression display the pattern of sperm first then oocytes as in wild-type. The opposite pattern, oocytes first then sperm, would not result in self fertility and thus would not be reproductively successful. The reason that these mutant combinations all display the wild-type pattern, instead of the oocyte then sperm pattern, is unclear and suggests that an additional level of sex determination pathway regulation remains to be uncovered

^c^ Mutually suppressing feminizing and masculinizing double-mutant hermaphrodites often have intersexual germ cells between the sperm and oocytes, unlike wild-type hermaphrodites

^d^ Embryos generated showed developmental arrest

Given the conservation of anatomy and reproductive physiology, an initial conversion from an ancestral *Caenorhabditis* female/male species to a hermaphrodite/male mode of reproduction may only require a genetic event that results in a transient increase in germline masculinizing activity in early larvae to produce sperm. As long as this change does not interfere with the higher level of feminizing activity (oogenesis) in late larvae/adults, self fertility would be possible. After the establishment of self fertility, there would likely be strong selection for additional genetic events that would optimize self-fertile brood size [70] and result in a clean transition from sperm to oocyte development so that wasteful intersexual gametes are not formed (Table 3). Thus, it is very likely that multiple genetic events now define the differences in the C. elegans and C. briggsae germline sex determination pathways.

In *C. elegans,* the relative levels of TRA-2 feminizing to FEM-3 masculinizing activity appear to be the major regulatory point for the sperm-then-oocyte pattern. There is no a priori reason for TRA-2 or FEM-3 to be the major focus of regulation to achieve hermaphroditism in *C. briggsae;* if one of these is the focus, then at least some of the regulation must differ between C. elegans and *C. briggsae,* given the absence of *fog-2* and the changed role of GLD-1. Since the last common ancestor of C. briggsae and C. elegans must have contained orthologs of 30 of 31 C. elegans sex determination genes, a change in the regulation of one or more of these genes might be responsible. Alternatively, since much of the regulation of C. elegans germline sex determination is by translational control, mutations in UTRs of mRNAs may result in new genes coming under the control of GLD-1 or another RNA sex determination gene regulator (Table 1). Additionally, duplication and divergence, analogous to what we have found for FOG-2 in *C. elegans,* may have resulted in a new gene being incorporated into the germline sex determination pathway. To move beyond speculation, the forward genetic analysis currently in progress (R. Ellis and E. Haag, personal communication) will be important for the identification of C. briggsae–specific genes, analogous to *fog-2,* that are necessary for self-fertile hermaphroditism.

## Materials and Methods

### 

#### Sex determination pathway conservation

Protein coding sequences of cloned C. elegans sex determination genes were obtained from Wormbase (http://www.wormbase.org; WormPep release 112). C. briggsae genomic sequence was obtained from The Sanger Institute (Cambridge, United Kingdom) or the Genome Sequencing Center (St. Louis, Missouri, United States), and protein sequences were obtained from either Wormbase or Ensemble (http://www.ensembl.org/; version 17.25.1). Best BLAST orthologs of C. briggsae sex determination proteins were obtained using C. elegans sex determination protein sequences as queries against C. briggsae predicted proteins and six-frame translated C. briggsae genomic sequence. C. briggsae proteins obtained at an e-value cutoff of 1 × 10^−50^ reciprocal best hits were recovered for 26 of 31 C. elegans proteins. NOS-1 and XOL-1 orthologs were identified at an e-value cutoff of 1 × 10^−20^ and were also reciprocal best BLAST hits between species. In each case a single reciprocal best hit was identified for each component of the sex determination pathway with the exception of FBF-1 and FBF-2, which returned the same best BLAST hit, and FOG-2. Searches of the non-redundant National Center for Biotechnology Information protein database (GenBank CDS+PDB+SwissProt+PIR+WormPep) with full-length FOG-2 as query revealed only weak similarity to the F-box motif for non–C. elegans or –C. briggsae sequences. Using the highly diverged C-terminal end of FOG-2, including a portion of the Duf38/FTH, or the GLD-1 interaction region of FOG-2 as query did not reveal any hits below an e-value of 0.01 in C. elegans or C. briggsae other than FOG-2 and FTR-1.

#### Identification of FTR family members

FTR family members are defined by the presence of an N-terminal F-box and C-terminal Duf38/FTH domain (FTR) [25]. C. elegans FTR family members were identified using FOG-2 as a query against WormPep release 112. Each potential FTR was scanned for an N-terminal F-box motif and C-terminal Duf38/FTH domain using the hidden Markov models (HMMs) for each domain (HMMER 2.3.2) [35]. Similarly, C. briggsae FTR family members were identified using FOG-2 as a BLAST query and HMMs. In *C. elegans, fog-2* (Y113G7B.5), *ftr-1* (Y113G7B.4), CE35646 (Y113G7B.1), CE24144 (Y113G7B.3), CE23289 (Y113G7B.6), and CE23288 (Y113G7B.7) are closely related and tightly linked on Chromosome 5. CE35646 was not included in later analysis because of a divergent N-terminal structure.

An FTR family also appears to be present and expanded in the obligate male/female species C. remanei based on the currently sequence assembly (Genome Sequencing Center, Washington University, St. Louis, Missouri, United States; 16 September 2004, BLASTn and tBLASTn; ftp://genome.wustl.edu/pub/seqmgr/remanei/plasmid_assembly). Our preliminary analysis suggests that closest FOG-2 homologs from C. remanei have diverged from C. elegans approximately to the same level as the FTR genes in *C. briggsae.* A comprehensive phylogenetic analysis to resolve the relationships between *C. elegans, C. briggsae,* and C. remanei FTR family members will await accurate C. remanei protein predictions and a complete C. remanei assembly.

#### Sequence alignments and analysis

Alignments were generated using CLUSTALW, and conserved residues were identified with the Lasergene MEGALIGN (DNASTAR, Madison, Wisconsin, United States) package and Dialign [71,72], which was also used to identify conserved regions for subsequent phylogenetic analysis. The best BLAST C. briggsae hit to each C. elegans FTR protein used in the phylogeny was included in order to identify any potential one-to-one orthologous pairs along the FOG-2 branch. Non-homologous N- and C-terminal extensions were trimmed, and extremely distant family members unlikely to be functional FOG-2 orthologs were excluded to avoid long branch attraction [47]. Phylogenetic inference was performed using the neighbor-joining (neighbor) program in the PHYLIP package (Phylogeny Inference Package version 3.5c; Department of Genetics, University of Washington, Seattle, Washington, United States) using the BLOSUM45 distance matrix. Trees with and without gaps were generated, and comparison revealed some differences in branching order, but only within the species. For the tree presented here, positions with gaps were excluded and all non-homologous or highly divergent sequences trimmed. The topology of the tree structure was tested by bootstrapping with 1,000 replicates and by analysis of the alignment using protpars from the PHYLIP package (a maximum parsimony method), which produced a tree with a similar branching order. Trees were processed using TreeView [73].

Codon-restricted alignments for K_a_/K_s_ calculation were generated using Se-Al (a sequence alignment editor by A. Rambaut, version 2; available at http://evolve.zoo.ox.ac.uk/software.html?id=seal) to modify CLUSTALW-aligned cDNA or predicted cDNA sequences, and all gaps and frame-shifted regions were removed. Sliding-window K_a_ and K_s_ estimates [74] were generated using DNASP (version 3) [52], and codon-based analysis was performed using PAML (codeml) [53] (HKY substitution model) to confirm the presence of codons under positive selection (95% confidence) within the sliding windows.

#### Worm culture and RNAi

C. elegans (N2, Bristol, United Kingdom) and C. briggsae (AF16) were obtained from the *Caenorhabditis* Genetics Center University of Minnesota, Minneapolis, Minnesota, United States. Cultures of both were maintained on Escherichia coli OP50 on NGM plates at 20 °C as previously described [75]. RNAi was performed by injection in C. elegans and C. briggsae essentially as described previously [54]. Double-stranded RNAs for species-specific *gld-1* and *fog-3* were generated by PCR amplification of cDNA with SP6 (5′) and T7 (3′) linkers, gel purified, sequenced, and used in RNA synthesis reaction using the appropriate Ambion kit (MEGAscript SP6 or T7; Austin, Texas, United States). Double-stranded RNAs were injected at 0.5 mg/ml into young adult N2 animals and F1 progeny collected 12–48 h post injection and matured to 24 h post L4 stage before gonads were dissected, fixed, and stained to score for abnormal phenotypes.

#### Staining

Dissection, antibody, and DAPI staining of C. elegans and C. briggsae gonads were performed essentially as previously described with fixation in 3% formaldehyde, 80% methanol, and 100 mM dibasic potassium phosphate [29,30]. Affinity purified rabbit polyclonal anti-GLD-1 antibodies were used at 1:50, and MSP mouse monoclonal antibody was used at 1:2,000, both with overnight incubation at room temperature (anti-MSP antibody was the kind gift of M. Kosinski and D. Greenstein, Vanderbilt University School of Medicine, Nashville, Tennessee, United States). Texas Red or Alexa488 secondary antibodies were used to detect staining, and DAPI was used visualize DNA morphology. Epifluorescent images were captured with a Zeiss (Oberkochen, Germany) Axioskop coupled to a Hamamatsu Photonics (Hamamatsu City, Japan) digital CCD camera, and processed with Photoshop 7.0 (Adobe, San Jose, California, United States). All image post-processing (brightness, contrast, pseudo-color, unsharp mask) was performed identically for each image.

#### Constructs and transformation

GLD-1 and FOG-2 yeast two-hybrid binding assays were performed as previously described [25] with the inclusion of 20 mM 3-amino-triazole. Progressive C-terminal deletions in FOG-2 and FTR-1/FOG-2 chimeric constructs were generated using PCR amplification of the appropriate coding sequences (FOG-2 full-length [327 aa], 318 aa, 299 aa, 263 aa, or exon 4 [251aa], or FTR-1 full-length [318 aa]) and cloned by recombination in yeast. In each case GLD-1 was used as bait in the pAS1 vector (DNA binding) and FOG-2 deletion constructs in the pACTII vector (activation). FOG-2 was found to exhibit low levels of auto-activation in the pAS1 (DNA binding) vector, so binding assays were performed in only one direction to avoid background and using high levels of 3-amino-triazole. The constructs were sequenced, and the Skp1-related F-box-binding protein SKR-1 (in pAS1) was used as a positive control for interaction [76,77].

## Supporting Information

Figure S1 Phylogenetic Relationships of 30 C. elegans and C. briggsae FTR Genes Closely Related to FOG-2 Presented as a Rectangular Phylogram A clear separation of C. elegans and C. briggsae FTR genes (C. briggsae is in grey shade) is suggested by the phylogeny. The branch containing FOG-2 and FTR-1 is in bold. Tree is unrooted, and branch lengths are proportional to divergence. Bar represents 0.1 substitutions per site. Bootstrap support for separation of C. elegans and C. briggsae sequences is indicated at the node (black dot) and at each node for the C. elegans FOG-2 branch.
(34.1 MB TIF).

Figure S2 Alignments of FTR-1 and FOG-2 C-Terminal Regions to Other Closely related C. elegans FTR Family Members (A) FTR-1 and FTR family alignment. Residues identical to FTR-1 are shaded black, and residues identical between all FTR family members tested are shaded red. Average pairwise identity to FTR-1 is 48%.
(B) FOG-2 and FTR family alignment. Residues identical to FOG-2 are shaded black, and residues identical between all FTR family members tested are shaded red. Average pairwise identity to FOG-2 is 22%.
(15.6 MB TIF).

Table S1 Analysis of Genes in the *fog-2* Cluster (59 KB PDF).

Table S2 Analysis of Genes Surrounding Y113G7B.11 in C. briggsae
(59 KB PDF).

## Abbreviations

DAPI: 4′,6′-diamidino-2-phenylindole hydrochloride
FTH: FOG-2 homology domain
FTR: *fog-2* related (F-box and FTH)
GFP: green fluorescent protein
HMM: hidden Markov model
K_a_: non-synonymous substitutions
K_s_: synonymous substitutions
L[number]: [number] larval
MSP: major sperm protein
RNAi: double-stranded-RNA-mediated interference
TGE: *tra-2* and GLI element
UTR: untranslated region
