# Supplementary figures and images for: fog-2 and the Evolution of Self-Fertile Hermaphroditism in Caenorhabditis
Source: PLoS Biol. 2004 Dec 28;3(1):e6. doi: 10.1371/journal.pbio.0030006 (PMC539060; doi:10.1371/journal.pbio.0030006)

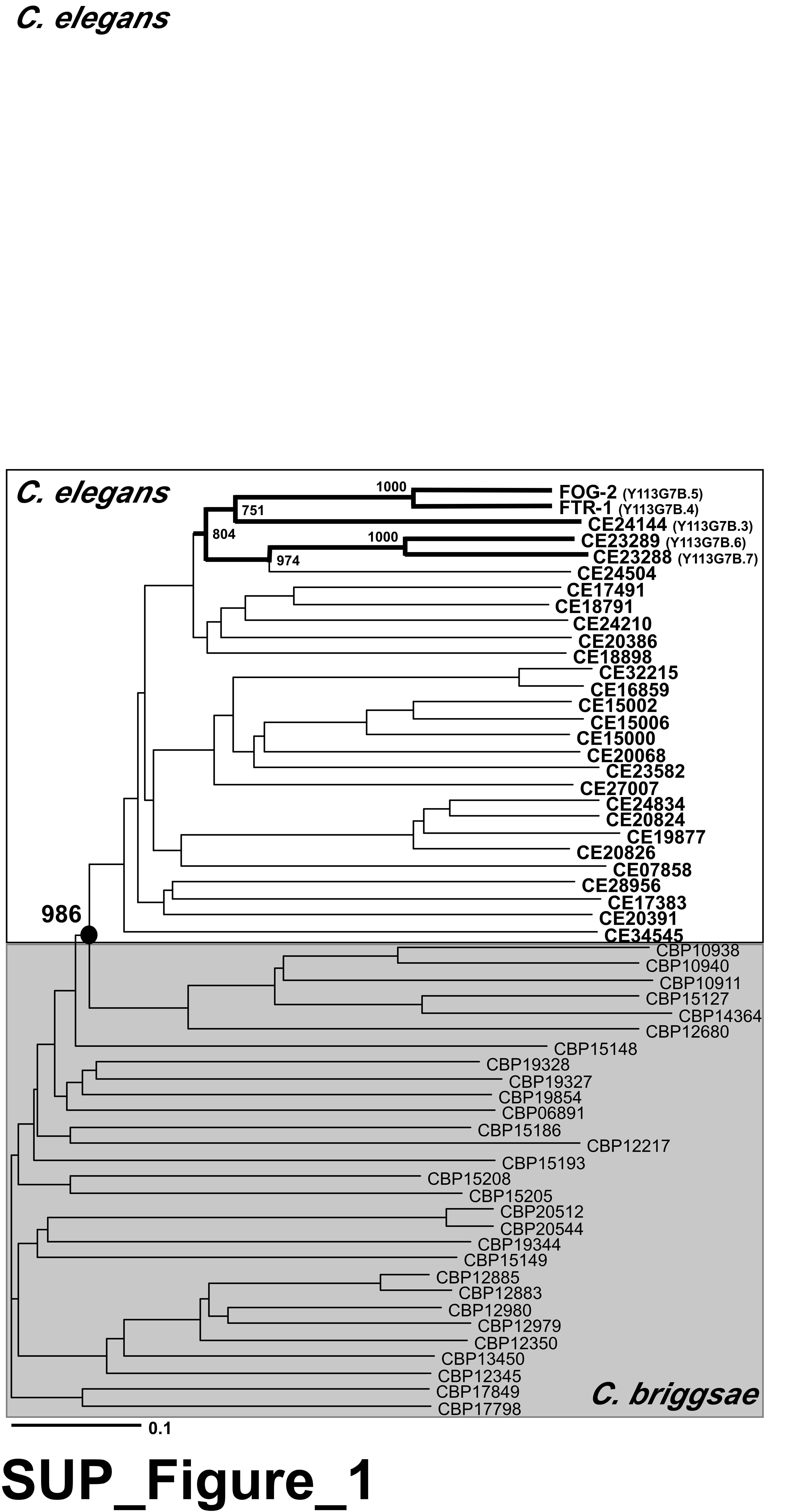

Supplement: Figure S1 — A clear separation of C. elegans and C. briggsae FTR genes (C. briggsae is in grey shade) is suggested by the phylogeny. The branch containing FOG-2 and FTR-1 is in bold. Tree is unrooted, and branch lengths are proportional to divergence. Bar represents 0.1 substitutions per site. Bootstrap support for separation of C. elegans and C. briggsae sequences is indicated at the node (black dot) and at each node for the C. elegans FOG-2 branch. (34.1 MB TIF). [file pbio.0030006.sg001.tif]

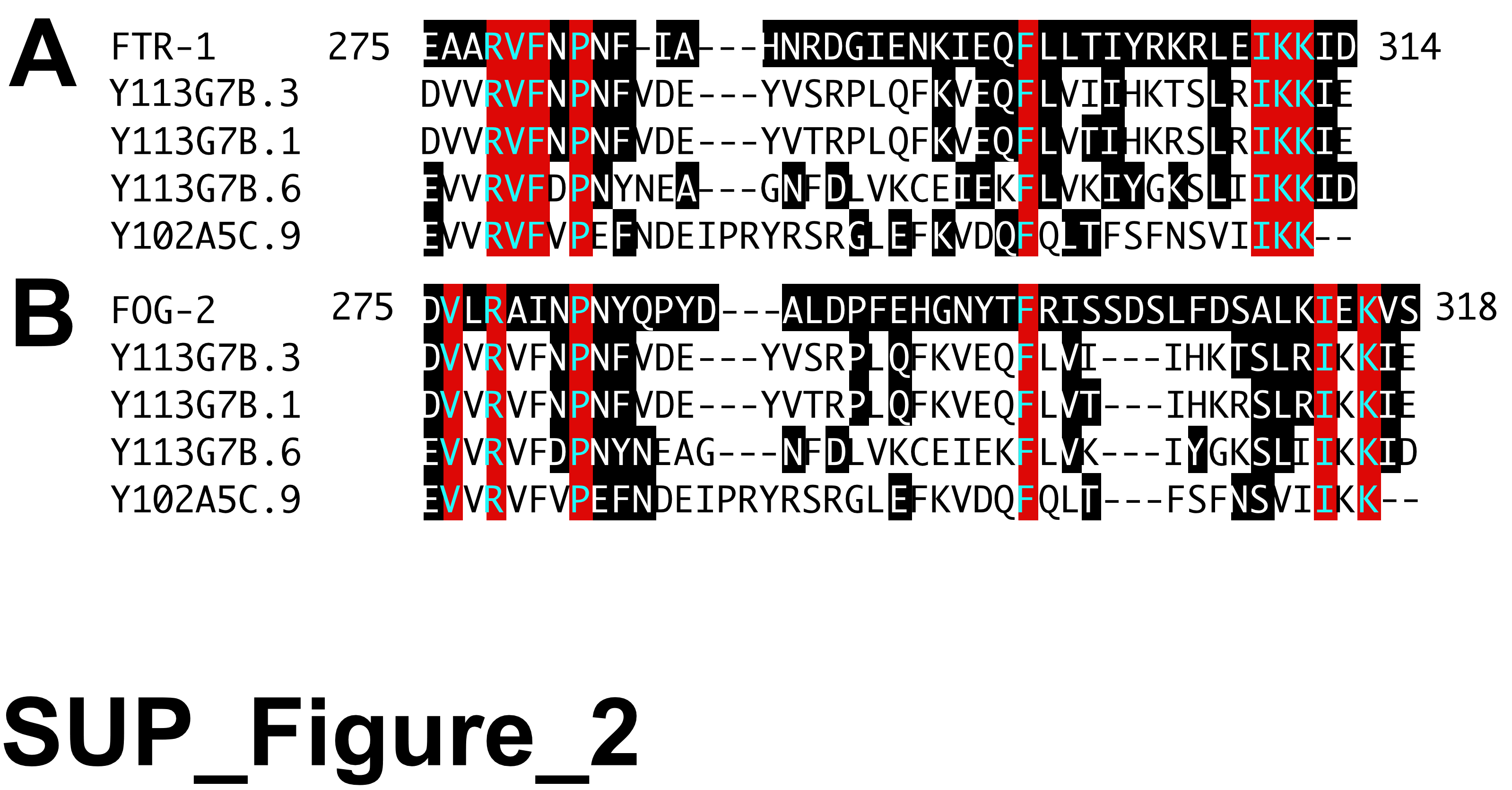

Supplement: Figure S2 — (A) FTR-1 and FTR family alignment. Residues identical to FTR-1 are shaded black, and residues identical between all FTR family members tested are shaded red. Average pairwise identity to FTR-1 is 48%. (B) FOG-2 and FTR family alignment. Residues identical to FOG-2 are shaded black, and residues identical between all FTR family members tested are shaded red. Average pairwise identity to FOG-2 is 22%. (15.6 MB TIF). [file pbio.0030006.sg002.tif]
