# Supplementary material for: fog-2 and the Evolution of Self-Fertile Hermaphroditism in Caenorhabditis
Source: PLoS Biol. 2004 Dec 28;3(1):e6. doi: 10.1371/journal.pbio.0030006 (PMC539060; doi:10.1371/journal.pbio.0030006)
Supplement: Table S1 — (59 KB PDF). [file pbio.0030006.st001.pdf]

Supplementary Table 1: Analysis of genes in the *fog-2* cluster

ID (WS130)	*C. elegans* Protein	*C. elegans* Position	Reciprocal best-BLAST	Family
Y113G7A.12	CE36453	V: 20142244-20144802	No	No Pfam definition
Y113G7A.13	CE24124	V: 20146197-20146685	No	No Pfam definition
F19B2.7	CE20699	V: 20147014-20153308	No	Peptidase S16, lon protease
F19B2.6	CE35496	V: 20156523-20159497	No	Transcription factor, DNA-binding
F19B2.5	CE20697	V: 20161352-20160645	No	DEAD/DEAH box helicase
F19B2.4	CE20696	V: 20165586-20167280	No	Protein of unknown function DUF216, 7TM related
F19B2.3	CE20695	V: 20170038-20168073	No	Protein of unknown function DUF1300
F19B2.2	CE20694	V: 20171803-20170818	No	Protein of unknown function DUF216, 7TM related
F19B2.1	CE20693	V: 20178740-20172000	No	Protein of unknown function DUF216, 7TM related
F19B2.8	CE33774	V: 20184676-20181840	No	Protein of unknown function DUF216, 7TM related
Y113G7B.1	CE35646	V: 20186879-20185707	No	FTR, diverged structure
Y113G7B.3	CE24144	V: 20190115-20188581	No	FTR
Y113G7B.2	CE23285	V: 20193243-20191460	No	7TM chemoreceptor
Y113G7B.4	FTR-1	V: 20194966-20196173	No	FTR
Y113G7B.5	FOG-2	V: 20197259-20198572	No	FTR
Y113G7B.7	CE23289	V: 20199647-20200756	No	FTR
Y113G7B.6	CE23288	V: 20201419-20202500	No	FTR
Y113G7B.8	CE23290	V: 20204177-20202565	No	F-box (lacks Duf38/FTH)
Y113G7B.9	CE23291	V: 20204667-20206400	No	Protein of unknown function DUF40
Y113G7B.12	CE23293	V: 20210776-20206798	No	Endonuclease/Exonuclease/phosphatase family
Y113G7B.11	**CE23292**	**V: 20213293-20211880**	**Yes(*)**	**No Pfam definition**
Y113G7B.14	CE23294	V: 20224832-20226826	No	DEAD/DEAH box helicase

ID entries are *C. elegans* gene identifiers from Wrombase. Reciprocal best-BLAST hits are indicated by “YES” or "NO" using WS130 and *C. briggsae* protein predictions from Wormbase. "Family" is assigned based on the Pfam HMM designation (http://pfam.wustl.edu/).

*See Supplementary Table 2
