# Supplementary material for: fog-2 and the Evolution of Self-Fertile Hermaphroditism in Caenorhabditis
Source: PLoS Biol. 2004 Dec 28;3(1):e6. doi: 10.1371/journal.pbio.0030006 (PMC539060; doi:10.1371/journal.pbio.0030006)
Supplement: Table S2 — (59 KB PDF). [file pbio.0030006.st002.pdf]

Supplementary Table 2: Analysis of genes surrounding Y113G7B.11 in *C. briggsae*

CBG	CBP	*C. briggsae* Position	*C. elegans* hit	*C. elegans* protein	*C. elegans* position	Reciprocal best-BLAST	Family
CBG05605	CBP01452	cb25.fpc0129:346195..349647	*Y38H6C.20*	CE19114	V: 20549098-20545344	Yes	Protein kinase domain
CBG05606	CBP07137	cb25.fpc0129:350745,352545	*Y38H6C.16*	CE19110	V: 20536523-20534536	Yes	Intradiol ring-cleavage dioxygenase
CBG05607	CBP15389	cb25.fpc0129:353302..356779	*Y38H6C.14*	CE19108	V: 20532220-20528074	Yes	No Pfam definition
CBG05609	CBP01455	cb25.fpc0129:361368..365960	*F26F2.7*	CE19819	V: 20579047-20588372	Yes	Eukaryotic membrane protein
CBG05612	CBP07139	cb25.fpc0129:369860..370785	*F46B3.3*	CE18686	V: 20601417-20604444	Yes	Transthyretin-like family
**CBG05618**	**CBP15391**	**cb25.fpc0129:389120..390340**	***Y113G7B.11***	**CE23292**	**V: 20213293-20211880**	**Yes**	**No Pfam definition**
CBG05616	CBP07142	cb25.fpc0129:382325..381617	*F46B3.4*	CE18687	V: 20606571-20605855	Yes	No Pfam definition
CBG05619	CBP07143	cb25.fpc0129:391827..390564	*Y38H6A.1*	CE19093	V: 20323779-20326545	Yes	No Pfam definition
CBG05620	CBP07144	cb25.fpc0129:401309..402397	*Y38H6A.3*	CE20231	V: 20343254-20346178	Yes	Transthyretin-like family
CBG05621	CBP01459	cb25.fpc0129:403782..402440	*K02E2.1*	CE18836	V: 20349551-20352956	Yes	No Pfam definition
CBG05622	CBP01461	cb25.fpc0129:406886..408422	*M01B2.7*	CE16268	V: 15261735-15260167	Yes	Rhodopsin-like GPCR superfamily
CBG05624	CBG01464	cb25.fpc0129:412874..414268	*F53F8.1*	CE17855	V: 20683574-20686821	Yes	Zinc finger, C2H2 type
CBG05625	CBG01467	cb25.fpc0129:415585..414417	*F53F8.4*	CE17858	V: 20689736-20688827	Yes	Alpha-macroglobulin
CBG05626	CBG07147	cb25.fpc0129:423877..422673	*F53F8.5*	CE17859	V: 20702088-20698610	Yes	SAM domain
CBG05629	CBG07149	cb25.fpc0129:435188..437023	*Y44A6C.1*	CE28852	V: 20711997..20714900	Yes	No Pfam definition

CBG = *C. briggsae* gene. CBP = *C. briggsae* peptide. CBG and CBP entries are from Wrombase. *C. elegans* hit and *C. elegans* protein entries are from Wormbase (WS130). Reciprocal best-BLAST hits are indicated by “YES” or "NO" using WS130 and *C. briggsae* protein predictions. "Family" is assigned based on the Pfam HMM designation (http://pfam.wustl.edu/).
